# Transcriptomic and Metabolomic Analyses Reveal Inhibition of Hepatic Adipogenesis and Fat Catabolism in Yak for Adaptation to Forage Shortage During Cold Season

**DOI:** 10.3389/fcell.2021.759521

**Published:** 2022-01-17

**Authors:** Juanshan Zheng, Mei Du, Jianbo Zhang, Zeyi Liang, Anum Ali Ahmad, Jiahao Shen, Ghasem Hosseini Salekdeh, Xuezhi Ding

**Affiliations:** ^1^ Key Laboratory of Veterinary Pharmaceutical Development, Ministry of Agricultural and Rural Affairs & Key Laboratory of Yak Breeding Engineering, Lanzhou Institute of Husbandry and Pharmaceutical Sciences, Chinese Academy of Agricultural Sciences, Lanzhou, China; ^2^ State Key Laboratory of Grassland Agro-Ecosystems, School of Life Sciences, Lanzhou University, Lanzhou, China; ^3^ Department of Systems Biology, Agricultural Biotechnology Research Institute of Iran, Agricultural Research, Education, and Extension Organization, Karaj, Iran; ^4^ Key Laboratory of Veterinary Pharmaceutical Development, Ministry of Agricultural and Rural Affairs, Lanzhou Institute of Husbandry and Pharmaceutical Sciences, Chinese Academy of Agricultural Sciences, Lanzhou, China

**Keywords:** forage shortage, yak, liver, energy metabolism, transcriptome, metabolomics

## Abstract

Animals have adapted behavioral and physiological strategies to conserve energy during periods of adverse conditions. Hepatic glucose is one such adaptation used by grazing animals. While large vertebrates have been shown to have feed utilization and deposition of nutrients—fluctuations in metabolic rate—little is known about the regulating mechanism that controls hepatic metabolism in yaks under grazing conditions in the cold season. Hence, the objective of this research was to integrate transcriptomic and metabolomic data to better understand how the hepatic responds to chronic nutrient stress. Our analyses indicated that the blood parameters related to energy metabolism (glucose, total cholesterol, low-density lipoprotein cholesterol, high-density lipoprotein cholesterol, lipoprotein lipase, insulin, and insulin-like growth factor 1) were significantly (*p* < 0.05) lower in the cold season. The RNA-Seq results showed that malnutrition inhibited lipid synthesis (particularly fatty acid, cholesterol, and steroid synthesis), fatty acid oxidation, and lipid catabolism and promoted gluconeogenesis by inhibiting the peroxisome proliferator-activated receptor (PPAR) and PI3K-Akt signaling pathways. For metabolite profiles, 359 metabolites were significantly altered in two groups. Interestingly, the cold season group remarkably decreased glutathione and phosphatidylcholine (18:2 (2E, 4E)/0:0). Moreover, integrative analysis of the transcriptome and metabolome demonstrated that glycolysis or gluconeogenesis, PPAR signaling pathway, fatty acid biosynthesis, steroid biosynthesis, and glutathione metabolism play an important role in the potential relationship between differential expression genes and metabolites. The reduced lipid synthesis, fatty acid oxidation, and fat catabolism facilitated gluconeogenesis by inhibiting the PPAR and PI3K-Akt signaling pathways to maintain the energy homeostasis of the whole body in the yak, thereby coping with the shortage of forages and adapting to the extreme environment of the Qinghai-Tibetan Plateau (QTP).

## Highlights

• Nutritional stress caused differential alterations of various hepatic metabolites, genes, and related pathways.
• The facilitated gluconeogenesis and fatty acid oxidation and reduced the fat catabolism by inhibiting the peroxisome proliferator-activated receptor signaling pathway and PI3K-Akt signaling pathway to cope with the shortage of forages.
• Cold season grazing inhibited the expression of genes responsible for *de novo* fatty acids synthesis (ACACA, ACACB, and FASN), fatty acid uptake (LPL, OLR1), FA desaturation (SCD), and FA transportation (LDLR), fatty acid oxidation (CPT1C), and lipolysis (FABP4).

## Introduction

The Qinghai-Tibetan Plateau (QTP), China’s largest and highest region, is a significant livestock production area and a global biodiversity hotspot (Jing et al., 2021). It is characterized by hypoxia, high altitude, short forage growing, and long-term cold season that extends from October to May, average temperature −5°C to −15°C (Hu et al., 2019). Yak (*Bos grunniens*), a unique livestock species found mostly at high altitudes (over 4,000 m) on the QTP, plays an important role in the daily lives of local herders by providing basic production and living materials such as animal-derived food, shelter, and fuel (Hu et al., 2019). As traditional grazing livestock, yaks graze without supplementary feeding all year round; consequently, they suffer from nutritional stress for a long time due to a shortage of forage during the long-term cold season. However, yaks have lived on the QTP for thousands of years and are well adapted to cope with the harsh conditions of the QTP. It is possible that the regulation of hepatic gene expression reduced metabolic rate, and energy requirements may be adjusted in response to harsh environment by changing the capacity of enzymes (Weber et al., 2017; Jing et al., 2020; Jing et al., 2021). The liver is the main metabolic site of glucose, fatty acids, and protein and plays an important role in regulating the composition of peripheral blood nutrients and the energy metabolism balance of the animal body (Costa et al., 2014; Rui, 2014; Jing et al., 2021). The warm season pastures on the QTP grow luxuriantly, and the supply is sufficient. Yaks, like other livestock, synthesize fatty acids through *de novo* lipogenesis (DNL), and long-chain fatty acids are contributed in hepatocytes for triglycerides (TG), phospholipids, or cholesterol esters (Rui, 2014). These complex lipids are stored in lipid droplets or secreted into the circulation as intestinally derived chylomicrons (CMs) and very low-density lipoprotein (VLDL) particles. The genes involved in lipid synthesis were down-regulated, whereas the pathways gluconeogenesis (Yu et al., 2016), fatty acid oxidation (Yu et al., 2016; Ren et al., 2019), and peroxisome proliferator-activated receptor A (PPAR-A) target genes (involved in lipolysis) (Laporta et al., 2014) were up-regulated in ruminants under nutritional deprivation. However, the recent study suggests that lipid catabolism, fatty acid synthesis, glucose intake, and fatty acid oxidation in yaks were all inhibited by regulating the AMPK (adenosine 5′-monophosphate-activated protein kinase) signaling over cold seasons (Xiong et al., 2020). Consequently, we speculate that the extreme long-term environment and nutritional stress caused by yak has formed a unique metabolic mechanism in the liver to help the host reduce energy consumption under the traditional grazing regime and maintain the body’s energy homeostasis to effectively cope with the long-term withered grass period. However, the regulating mechanism of hepatic metabolism in yaks remains unknown under grazing conditions in cold seasons.

Lipid catabolism is a biochemical process that begins with fatty acid oxidation and ends with glucose production. It is influenced by nutrients, key hormones, transcriptional factors, and lipolytic enzymes (Li Y. et al., 2020). The genes such as stearoyl-CoA desaturase (SCD), fatty acid synthase (FASN), lipoprotein lipase (LPL), insulin (INS), acetyl-CoA carboxylase (ACC), and ACSL (acyl-CoA synthetase long-chain), all of which take part in lipogenesis, lipolysis, fatty acid transport, and cholesterol metabolism (Zhong et al., 2020). It is reported that the activity of LPL and INS in yak was sensitive as a result of alterations in nutritional conditions and seasons (Ding et al., 2012). Therefore, further research on the expression and regulation of genes is necessary for different seasons. Studies have revealed that the regulation of hepatic gene expression plays a crucial role in hepatic metabolism by changing the capacity of enzymes in relevant metabolic pathways (Pamela A. Alexandre et al., 2015; Ren et al., 2019). Furthermore, results of the recent studies suggest that the genes involved in hepatic lipid synthesis (ACACA, FASN, LPL, SCD1, FADS1, and FADS2) were down-regulated over nutritional deficiency (Kaufmann et al., 2012; Laporta et al., 2014; Vailati Riboni et al., 2015). Our previous study in yak also indicated the expression of genes FASN, LPL, ACACA, PPARγ, and SREBP-1c with increasing energy levels, whereas there was low expression of HSL, CPT-1, and ATGL (Yang et al., 2020). However, most of the current research is based on specific functional genes and enzymes, and the regulation of target pathways in the hepatic response of yak is less well understood under grazing conditions. The hepatic response in ruminants has been performed to reveal the pathways such as glycolysis or gluconeogenesis, fatty acid biosynthesis, extracellular matrix–receptor interaction, protein digestion and absorption, and cholesterol homeostasis, which play an essential role in nutritional metabolism (Ren et al., 2018; Lu et al., 2019; Ren et al., 2019; Yu et al., 2019). Little information has been published concerning gene expression and target pathways in hepatic energy metabolism in yaks under natural grazing. In addition, knowledge about hepatic energy metabolism in the cold season may help explore yak’s modulatory molecular mechanisms to adapt to the long-term withered grass period.

In recent years, the emerging “omics” technologies, including transcriptomics, metabolomics, and proteomics, have greatly accelerated research on the interactions between nutrients and diet in a biological system (Laporta et al., 2014; Alexandre et al., 2015). Transcriptomic and metabolomics have become the focus of nutrition research. The modulatory mechanisms of hepatic metabolism can be revealed by integrating transcriptomics and metabolomics (Yu et al., 2019). There have been extensive studies that demonstrated marked changes in ruminant’s hepatic transcriptome and metabolite profiles due to changes in different feeding regimes, particularly genes, metabolites, and pathways related to lipid metabolism (Laporta et al., 2014; Sun et al., 2018). So far, however, studies for hepatic energy metabolism in yaks based on transcriptomics and metabolomics in the nutrition stress state are rarely reported.

Therefore, we hypothesize that yak has developed a core metabolic mechanism that helps the host to adapt to the harsh environment and long-term withered grass period of the Qinghai-Tibet Plateau. In the present study, we integrated transcriptomic, metabolomic and animal serum parameters to provide new insights into the hepatic metabolism and nutritional strategies in yak for adaptation to harsh environment.

## Materials and Methods

### Study Site

All yaks were grazing on the natural alpine pasture under a traditional farming system on the Qinghai-Tibetan Plateau with free access to water. Six grass species predominate the natural vegetation, namely, *Kobresia pygmaea*, *Elymus nutans*, *Kobresia humilis*, *Kobresia capillifolia*, *Stipa purpurea*, and *Potentilla acaulis*. This study was conducted at Haibei Demonstration Zone of Plateau Modern Ecological Animal Husbandry Scientific and Technology in Haibei Prefecture, Haiyan County (36° 44′ to 39° 05′ N″ 130 97° 17′ to 102° 41′ E), Qinghai Province, China. This area has an average altitude of more than 3,000 m above sea level, an average annual temperature of 0.45°C, average annual precipitation of 277.8 to 499.5 mm, and a dry, cold winter climate. Alpine and subalpine herbage meadows in the region are important for yak production, with seasonal migration between different grazing areas. Typically, transhumance farming defined by switching between different seasonal pasture sites is practiced, with cold season and warm season pastures belonging to one type but being divided by fences as different seasonal pastures (Ding et al., 2012).

### Animals and Management

A total of twelve 4-year-old healthy adult female yaks with initial average body weight (BW) 270 ± 10.6 kg were selected and randomly divided into a warm-season group (YW) and cold season (YC) group, with six yaks each group, and properly marked with ear tags for identification. The warm season group was grazed on the natural alpine pasture in the Qinghai-Tibetan Plateau from May to September, whereas the yaks in cold season were grazed from October to the following April, which was thought to correspond to the increasing stage of alpine grasses and changes in the nutritional status of yaks in a whole production year. Moreover, the experimental procedures in animal care in this study were approved by the Animal Care and Use Committee of Lanzhou Institute of Husbandry and Pharmaceutical Sciences, CAAS, and China [SYXK-2018–0011]. Meanwhile, all yaks care procedures were consistent with the Guide, local animal welfare laws for the Care and Use of Laboratory Animals (Gansu Province Animal Care Committee, Lanzhou, and China).

The forage samples were collected with the previous method in mid-month; vegetation samples were cut within a 10 × 10-cm quadrat frame. Each plot was randomly sampled for 30 small samples, mixed into a large sample, and each plot was repeated five times. The feces of the yaks were collected each month; in each plot, 15 fresh feces were randomly selected as small samples, mixed into a large sample, with five replicates for each plot. The dry matter (DM), crude protein (CP), neutral detergent fiber (NDF), ether extract (EE), and acid detergent fiber (ADF) were measured according to previous methods (Van Soest et al., 1991). The nutritional composition of pasture herbage is listed in Supplementary Table S1. The DM intake (DMI), metabolic energy intake, DM digestibility, CP digestibility, NDF digestibility, and ADF digestibility was measured by previous studies (Oba and Allen, 1999). The results are presented in Supplementary Table S2.

### Sample Collection

Following fasting for 8 h, blood samples were collected from the jugular vein into EDTA tubes and chilled on ice. After centrifugation (2,000×*g*, 4°C, and 20 min) of the blood samples, the plasma was divided into portions and frozen at –20°C and used for measurement of glucose, INS, total cholesterol, nonesterified fatty acids, triglyceride, and so on. The liver tissue was collected after slaughtering and immediately washed with 0.90% NaCl solution and stored in liquid nitrogen for gene expression and metabolome analysis.

### Measurement of Blood Biochemical and Hormonal Parameters

Triglyceride (TG), cholesterol (CH), high-density lipoprotein cholesterol (HDL-C), low-density lipoprotein cholesterol (LDL-C), lactate dehydrogenase (LDH), glucose, nonesterified fatty acids (NEFAs), and creatine kinase (CK) levels, of plasma, were determined with an automatic biochemical analyzer (TG, Cat #: A110-1-1; CH, Cat #: A111-1-1; HDL-C, Cat #: A020-1-2; LDL-C, Cat #: A113-1-1; LDH, Cat #: A110-1-1; NEFAs, Cat #: A042-1; CK, Cat #: A032-1-1; Shenzhen Mindray Bio-Medical Electronics Company Limited, Shenzhen, and China).

The concentrations of FAS, growth hormone (GH), INS-like growth factor 1 (IGF-1), INS-like growth factor 2 (IGF-2), glutathione peroxidase (GSH-PX), LPL, INS, malondialdehyde (MDA), superoxide dismutase (SOD), and total antioxidant capacity (T-AOC) were measured according to the manufacturer’s instructions (FAS, bovine FAS ELISA KIT, Cat #: ZC-50645; GH, bovine GH ELISA KIT, Cat #: ZC-50478; IGF-1, bovine IGF-1 ELISA KIT, Cat #: ZC-50596; IGF-2, bovine IGF-2 ELISA KIT, Cat #: ZC-54217; GSH-PX, bovine GSH-PX ELISA KIT, Cat #: ZC-54011; LPL, bovine LPL ELISA KIT, Cat #: ZC-50640; INS, bovine INS ELISA KIT, Cat #: ZC-50594; MDA, bovine MDA ELISA KIT, Cat #: ZC-50180; SOD, bovine SOD ELISA KIT, Cat #: ZC-50189; T-AOC, bovine T-AOC ELISA KIT, Cat #: ZC-53993; Chengdu Li Lai Biotechnology Company Limited, Chengdu, and China).

### Metabolomic Profiling Analysis

The hepatic metabolome was analyzed using a Vanquish UHPLC system (Thermo Fisher) coupled with an Orbitrap Q Exactive HF-X mass spectrometer (Thermo Fisher). Samples were separated on a Hyperil Gold column (100 × 2.1 mm, 1.9 μm) at a 0.2 mL/min flow rate. The eluents for the positive polarity mode and the negative polarity mode were eluent A (0.1% FA in water, 5 mM ammonium acetate, pH 9.0) and eluent B (methanol). The solvent gradient was as follows: 1.5 min, 2% B; 12.0 min, 2%–100% B; 14.0 min, 100% B; 14.1 min, 100%–2% B; 16 min, 2% B. Q Exactive HF-X mass spectrometer was performed in positive/negative polarity mode with a spray voltage of 3.2 kV, the capillary temperature of 320°C, and sheath gas and aux gas flow rate of 35 and 10 arb, respectively.

Progenesis QI (Waters Corporation, Milford, MA, USA) data processing software was used to identify metabolites. The metabolic alterations among experimental groups were visualized by principal component analysis (PCA) and (orthogonal) partial least-squares discriminant analysis (O) PLS-DA, after data preprocessing by mean centering (Ctr), and Pareto variance (Par) scaling, respectively. Metabolites were identified with variable importance in the projection (VIP) values larger than 1.0 and *p* values less than 0.05, and FC less than or equal to 0.67 or FC larger than or equal to 1.5 were considered differential metabolites. The metabolic pathways and metabolite set enrichment analysis were analyzed using MetaboAnalyst 5.0 (https://www.metaboanalyst.ca/).

### RNA Preparation and Transcriptome Sequencing

Liver tissues of yak were used to total RNA extraction using TRIzol reagent (Invitrogen, Carlsbad, CA, USA) following the manufacturer’s protocol. The Agilent 2,100 Bioanalyzer was performed to detect RNA integrity (Agilent Technologies, Santa Clara, CA, USA). All samples with RNA Integrity Number ≥7 were subsequently analyzed. The mRNA was established sequencing libraries using TruSeq RNA Sample Preparation Kit (Illumina, San Diego, CA, USA). Then cDNA libraries were sequenced on the Illumina sequencing platform (HiSeqTM 2,500), which generated paired-end reads of 125 bp/150 bp. FPKM (Roberts et al., 2011) value of each gene was calculated using cufflinks (Trapnell et al., 2010), and the htseq count was used to obtain the read counts of each gene (Anders et al., 2015). The differential expression genes (DEGs) between YW and YC groups were detected using DESeq (Anders and Huber, 2012) R package. The false discovery rate (FDR) <0.05 and the absolute value of the log_2_ (fold change) with FPKM ≥1 were used as the threshold for significantly different expression (Liu et al., 2021). Hierarchical cluster analysis of DEGs was performed to explore gene expression patterns. Gene Ontology (GO) and Kyoto Encyclopedia of Genes and Genomes (KEGG) (Kanehisa et al., 2007) pathway enrichment analysis of DEGs were respectively performed using R based on the hypergeometric distribution. GO terms and the pathways of KEGG with FDR <0.05 were considered remarkably enriched. The raw data of transcriptome sequencing for livers of yaks in YC and YW groups have been deposited in the National Center for Biotechnology Information (NCBI) at Sequence Read Archive with the accession number PRJNA756146.

### Real-Time Quantitative PCR

Real-time quantitative reverse transcriptase–polymerase chain reaction (qRT-PCR) assay was performed as described in our previous study (Yang et al., 2020). The β-actin was used as a reference gene to normalize gene expression. Primers used for qRT-PCR are listed in Supplementary Table S3. The qRT-PCR was performed in triplicate reactions to determine relative mRNA levels using SYBR® Premix Ex Taq™ II (TaRaKa, Dalian, and China). Fold change was calculated for each candidate gene, and sample was calculated by means of the formula the $2^{-\text{ΔΔCt }}$ (Schmittgen and Livak, 2008).

### Statistical Analysis

Data are presented as means ± SEM. All statistical analyses were performed by the one-way analysis of variance test, and the differences were compared using Tukey multiple-comparisons test, and *P* < 0.05 was considered to be statistically significant. Moreover, integrative analysis of DEGs and differential metabolites that involved hepatic energy metabolism was performed by MetaboAnalyst 5.0 (https://www.metaboanalyst.ca/). Pearson correlation algorithm was used to calculate the associations between discriminant gene expression and metabolites, when |*r*| > 0.60 and *p* < 0.05 were considered as a significant correlation.

## Results

### Serum Biochemical Parameters

Live BW fell through the whole winter as food accessibility decreased with decreasing above-ground herbage mass. Still, during the warm season, compensatory growth led to early and increasing benefits for mitigating body mass loss over the winter. The levels of GLU, CH, and TG were significantly higher in the YW group than those in the YC group (*p* = 0.044, *p* = 0.003, and *p* = 0.022; Table 1), indicating that the energy produced by glucose from forage cannot maintain the energy homeostasis of the body in the case of the shortage of forage. The yak needs to consume higher levels of GLU to maintain normal physiological function. Conversely, the concentration of NEFAs and CK in the YC group was significantly higher than that in the YW group (*p* = 0.012, *p* = 0.015), whereas differences were not statistically significant in the concentration of LDH (*p* > 0.05). Similarly, no significant differences in FAS, HSL, and IGF-2 were observed between the considered groups (*p* > 0.05; Table 2). However, the levels of LDL-C, HDL-C, IGF-1, INS, and LPL tended to be higher in warm season than those in cold season (*p* = 0.006, *p* = 0.025, *p* = 0.012, *p* = 0.002, and *p* = 0.020), whereas GH concentration was higher for the YC group than the YW group (*p* = 0.018) (Tables 1, 2). In addition, compared with the YC group, there was an increasing trend but no significant differences in serum antioxidant index, including T-AOC, GSH-PX, MDA, and SOD in the YW group (*p* > 0.05; Table 3)

**TABLE 1:** The effects of different seasons on serum biochemical parameters.

Item	Treatment		SEM	*p* value
	YC	YW		
TG (mmol/L)	0.18^b^	0.30^a^	0.10	0.022
LDH (U/L)	839.14	993.62	91.92	0.433
GLU (mmol/L)	3.68^b^	5.24^a^	0.52	0.044
LDL-C (mmol/L)	0.16^b^	0.41^a^	0.05	0.006
HDL-C (mmol/L)	1.04^b^	1.71^a^	0.16	0.025
CH (mmol/L)	1.11^b^	2.21^a^	0.22	0.003
CK (U/L)	742.50^a^	171.32^b^	116.74	0.015
NEFAs (µmol/L)	835.90^a^	545.64^b^	58.24	0.012

Note: TG, triglycerides; LDH, lactate dehydrogenase; GLU, glucose; LDL-C, low-density lipoprotein cholesterol; HDL-C, high-density lipoprotein cholesterol; CH, cholesterol; CK, creatine kinase; NEFAs, nonesterified fatty acids.

**TABLE 2:** The effects of different seasons on serum hormone of the yak.

Item	Treatment		SEM	*p* value
	YC	YW		
FAS (nmol/L)	2.93	1.94	0.33	0.145
GH (ng/mL)	3.66^a^	2.36^b^	0.42	0.018
HSL (ng/mL)	1.14	1.74	0.21	0.173
IGF-1 (ng/mL)	48.21^b^	55.15^a^	2.70	0.012
IGF-2 (ng/mL)	4.45	7.11	0.97	0.185
INS (mIU/L)	3.36^b^	5.10^a^	0.68	0.002
LPL (U/L)	96.31^b^	148.72^a^	13.96	0.020

Note: FAS, fatty acid synthetase; GH, growth hormone; HSL, Hormone-sensitive fatty lipase; IGF-1, insulin-like growth factor 1; IGF-2, insulin-like growth factor 2; INS, insulin; LPL, lipoprotein lipase.

**TABLE 3:** The effects of different seasons on plasma antioxidant index of the yak.

Item	Treatment		SEM	*p* value
	YC	YW		
T-AOC (U/mL)	9.09	5.95	1.19	0.206
GSH-PX (ng/mL)	970.15	523.30	129.47	0.082
MDA (nmol/mL)	2.04	1.36	0.25	0.181
SOD (ng/mL)	1.83	1.07	0.23	0.104

Note: T-AOC, bovine total antioxidant capability; GSH-PX, glutathione peroxidase; MDA, malondialdehyde; SOD, superoxide dismutase.

### Metabolomic Profiling

**FIGURE 1:**
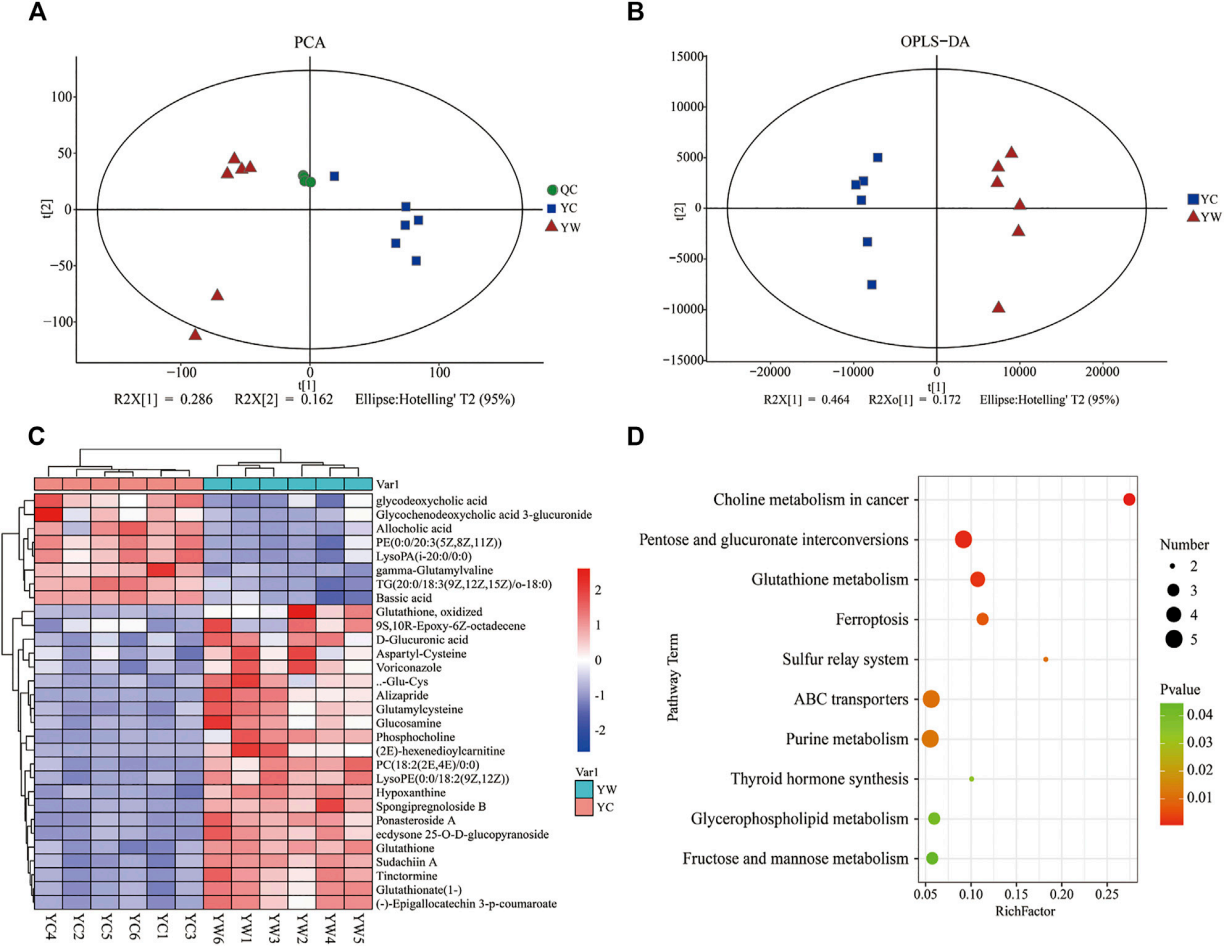
Hepatic metabolic profiles in YW/YC group. **(A)** All samples' principal component analysis (PCA) (at the metabolites level). **(B)** Orthogonal partial least-squares discriminant analysis (OPLS-DA). **(C)** Heat map of differential metabolites in YW/YC group. The abscissa indicates the sample name, and the ordinate indicates the differential metabolite. The color from blue to red indicates the abundance of expression of metabolites from low to high; that is, the redder indicates the higher expression abundance of differential metabolites. **(D)** Kyoto Encyclopedia of Genes and Genomes (KEGG) pathway terms enriched by metabolites of liver between YW and YC yaks. *X* axis means rich factor (rich factor = DEGs enriched in the pathway ÷ background genes in the pathway). *Y* axis represents the KEGG pathway terms. The color of roundness represents *p* value. The area of roundness represents the number of DEGs enriched in this pathway.

An untargeted metabolomics approach studied the specific impact of seasonal changes in external conditions, especially food availability, and on metabolomic profiling. Based on the extracted ion peaks, differential metabolites in yak liver were discriminated by the scatterplots, including the quality control samples (Figure 1A), which demonstrated the precision and repeatability of the untargeted liquid chromatography–mass spectrometry (LC-MS) detection. A total of 4,953 metabolites (3,311 in positive mode and 1,642 in negative mode) were detected and used for multivariate analysis of hepatic metabolites (Supplementary Figure S1). The OPLS-DA for the two groups reveals a clear distinction (Figure 1B); cumulative variation modeled in the component in the Y matrix (R^2^Ycum) was 0.986, and the cumulative estimate of the predictive ability of the model (Q2cum) was 0.957, which was validated by the permutation analysis (Q2=−0.493). Importantly, 306 main metabolites (213 up-regulated and 93 down-regulated) were considerably significantly altered from each other, especially under the different seasons (VIP > 1, *p*<0.05, and FC ≤ 0.67 or ≥1.5; Supplementary File S1).

To show the expression differences of metabolites in different samples more intuitively, we performed Hierarchical Clustering on all significantly different metabolites (*p* < 0.05) and conducted a visual analysis of differential metabolite expression levels based on VIP value. We found that metabolites had a significant difference in the YC and YW groups from in Figure 1C. And results showed that the YC group contained lower contents of the expression abundance of metabolites YC (*p* < 0.05), including phosphatidylcholine (PC) (18:2 (2E, 4E)/0:0), glutamylcysteine, glutathione, Lyso PC (18:1 (11Z)), phosphocholine, and Glu-Cys. In comparison, the YW group accumulated higher contents of glycochenodeoxycholic acid 3-glucuronide, TG (20:0/18:3 (9Z, 12Z, 15Z)/o-18:0), and allocholic acid. These results indicated that nutritional stress increased oxidative stress, reduced lipid transport metabolism, and boosted VLDL synthesis. In addition, KEGG pathway enrichment analysis was performed to screen the differential pathways in the liver of yaks with the effect of malnutrition (Figure 1D). The results revealed that the most abundant metabolites were mainly enriched in pentose and glucuronate interconversions, glutathione metabolism, ABC transporters. Meanwhile, glycerophospholipid metabolism and fructose and mannose metabolism were similarly enriched.

### Analysis of Different Expression Genes

**FIGURE 2:**
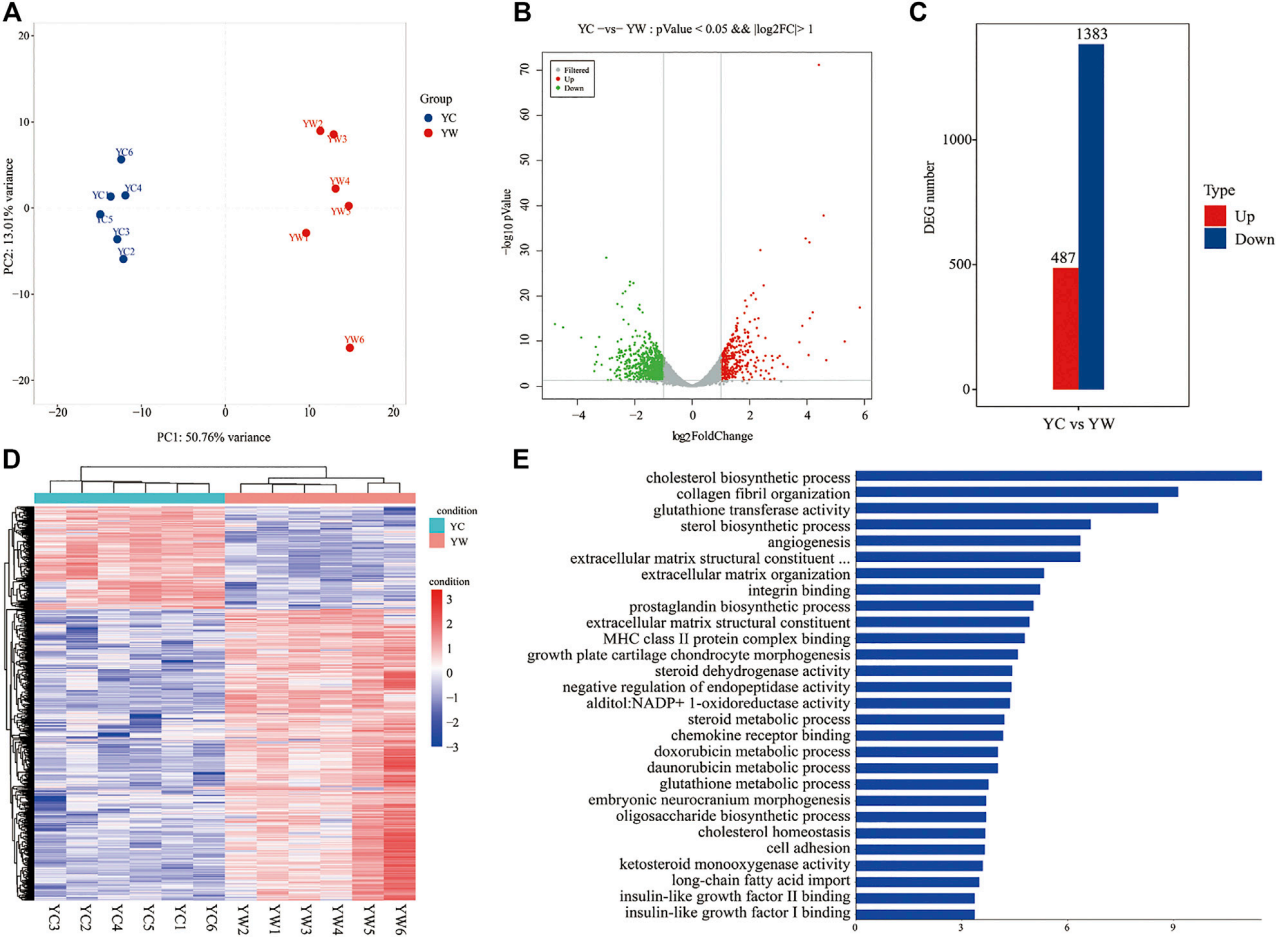
Transcriptomic comparisons of liver between YW and YC yaks. **(A)** All samples principal component analysis (PCA) (at the genes level). **(B)** Histogram of DEGs in liver of YW and YC yaks. **(C)** The volcanic map of DEGs in liver of YW and YC groups. Gray was the nonsignificantly different gene; red and green were the significantly different genes. The *X* axis represents log_2_ fold change, and the *Y* axis represents −log_10_ *p* value. **(D)** Heat map for hierarchical cluster analysis of DEGs between samples. Red: up-regulated genes; blue: down-regulated genes. **(E)** Gene Ontology (GO) analysis of the DEGs in liver of yaks. The top 30 GO terms with the lowest FDR in molecular function and biological process are shown, respectively. *Y* axis represents GO terms, and *X* axis represents the −log_10_ (*p* value).

To obtain a global view of the hepatic transcriptome responses in yak under different seasons, the hepatic transcriptomes were analyzed by comparative RNA-Seq. cDNA libraries were constructed using total RNA isolated from the same experimental materials as metabolic profiling. Using an Illumina HiSeq 2,500 sequencer, we obtained approximately 52.90 and 50.76 million high-quality clean reads from YW and YC groups, respectively. For each sample, 94.71% and 94.99% of reads could be mapped to the YW and YC yak reference genome, respectively, in which 90.54% and 91.10% aligned with unique genes unambiguously (Table 4). In total, 10,955 and 10,296 expressed genes (average FPKM >1) were detected in the livers of YW and YC yaks, respectively. The PCA approach showed that the samples were grouped closely, respectively (Figure 2A). DEGs were screened via DEGSeq with an FDR <0.05 and the absolute value of the log_2_ (fold change) with an FPKM ≥1. We identified 1,870 DEGs (487 up-regulated and 1,383 down-regulated) from the total of 17,724 genes (Figure 2B, C; DEG list is provided in Supplementary File S2). A heat map for hierarchical cluster analysis of DEGs between the two groups was more intuitive to support the view (Figure 2D).

**TABLE 4:** Summary of the sequencing reads alignment to the reference genome.

Statistics term	YW	YC
Clean reads	50.76	52.90
Total reads	50,760,057	52,896,499
Total mapped reads	94.99%	94.71%
Uniquely mapped	91.0%	90.54%
Nonsplice reads	49.39%	50.28%
Splice reads	41.70%	40.26%
Reads mapped in proper pairs	87.66%	87.11%

### GO Enrichment and Pathway Analysis of DEGs

GO enrichment analysis and the terms of top30 DEGs indicated that the energy metabolism and glutathione metabolism processes dominated the biological processes between the different seasons (FDR <0.05; Figure 2E). The GO terms of glutathione transferase activity and glutathione metabolic process were related to the glutathione metabolism. Notably, the GO terms of cholesterol biosynthetic process, sterol biosynthetic process, cholesterol homeostasis, IGF-2 binding, long-chain fatty acid import, and IGF-1 binding were closely related to energy metabolism.

**FIGURE 3:**
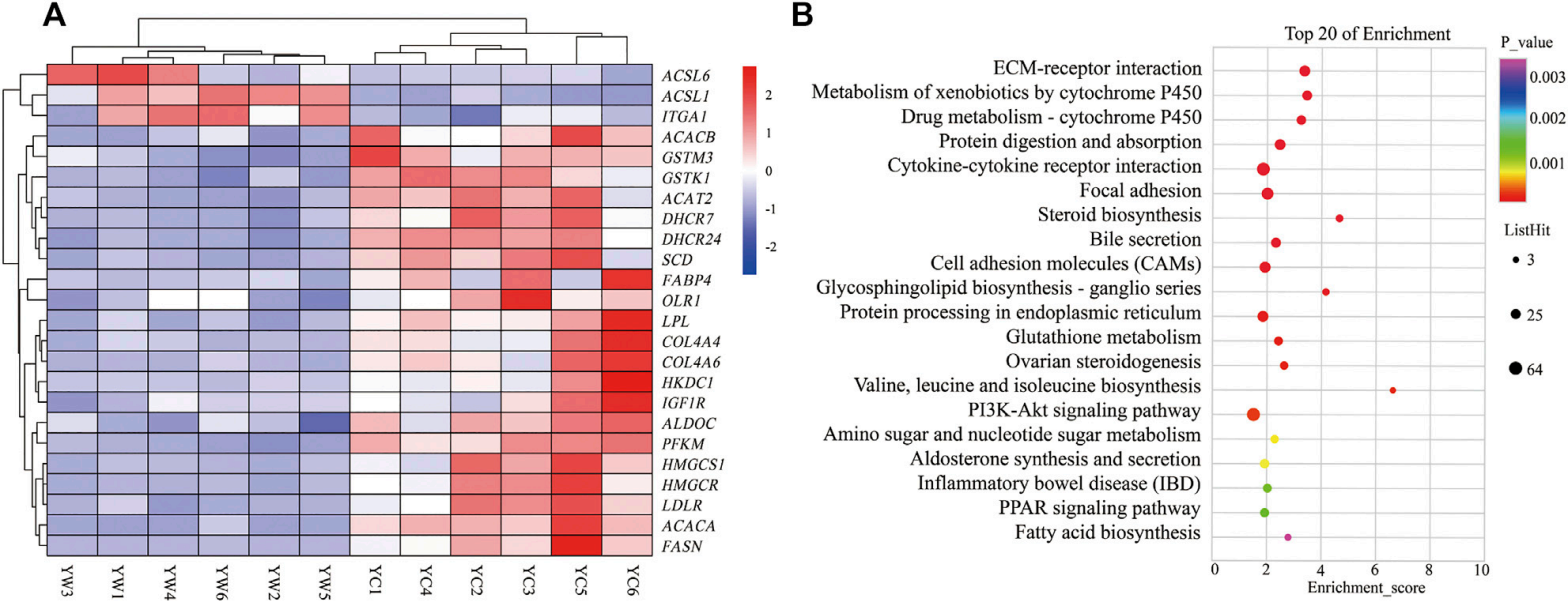
Heat map and KEGG pathways enrichment for DEGs. **(A)** Heat map of DEG-related hepatic energy metabolism. Red: up-regulated genes; blue: down-regulated genes. **(B)** KEGG pathways enrichment for DEGs. YC group versus YW group.

Furthermore, the KEGG analysis remarkably enriched 50 pathways for the identified DEGs (FDR <0.05; Figure 3B). ECM–receptor interaction (30 genes, adjusted *p* = 8.57 × 10^–9^), focal adhesion (47 genes, adjusted *p* = 2.53 × 10^–5^), protein digestion and absorption (34 genes, adjusted *p* = 9.50 × 10^–5^), metabolism of xenobiotics by cytochrome P450 (21 genes, adjusted *p* = 2.18 × 10^–6^), drug metabolism—cytochrome P450 (19 genes, adjusted *p* = 8.94 × 10^–6^), and glutathione metabolism (14 genes, adjusted *p* = 0.004) were significantly enriched. Typically, the expression levels of the genes including THBS1, COL4A4, SLC38A2, COL4A3, GSTK1, and GSTM3 were remarkably lower expressed in the liver of YC yaks compared with YW yaks, whereas ITGA1 had a higher expression. The bile secretion was significantly enriched with 23 genes (adjusted *p* = 0.0001), in which LDLR, HMGCR, and ABCB11 were highly expressed in YW yaks than YC yaks. Meanwhile, there were nine DEGs enriched in ovarian steroidogenesis (adjusted *p* = 0.004) and 10 DEGs enriched in steroid biosynthesis (adjusted *p* = 8.94 × 10^–6^). Mainly, including LDLR, IGF1R, FDFT1, and DHCR7 were weakly expressed in YC yaks. Interestingly, PI3K-Akt signaling pathway (53 genes, adjusted *p* = 0.004) were also enriched. Notably, the PPAR signaling pathway (16 genes, adjusted *p* = 0.01) and fatty acid biosynthesis (5 genes, adjusted *p* = 0.03) were significantly enriched. Typically, ACSL6 and ACSL1 had higher expression in YC yaks, whereas FABP4, SCD, HMGCS1, SLC27A2, HKDC1, LPL, OLR1, ACACA, ACACB, and FASN had lower expression (Figure 3A).

### qRT-PCR Validation of Functional Gene Expression

To validate the reliability gene expression from RNA-Seq, the transcriptome levels of 10 genes (ACACB, ALDOC, ACSL1, ACAT2, HK2, ACSL6, SEC61A1, GMPPB, SDS, and HKDC1) among the DEGs were determined by qRT-PCR with three replicates (Supplementary Figure S2). Of these, the expression trends were consistent with those obtained in the RNA-Seq analysis. In addition, the RNA-Seq and qRT-PCR results demonstrated that the data could be used to assess the up-regulation and down-regulation of gene expression.

### Integrative Analysis of Transcriptome and Metabolome

**FIGURE 4:**
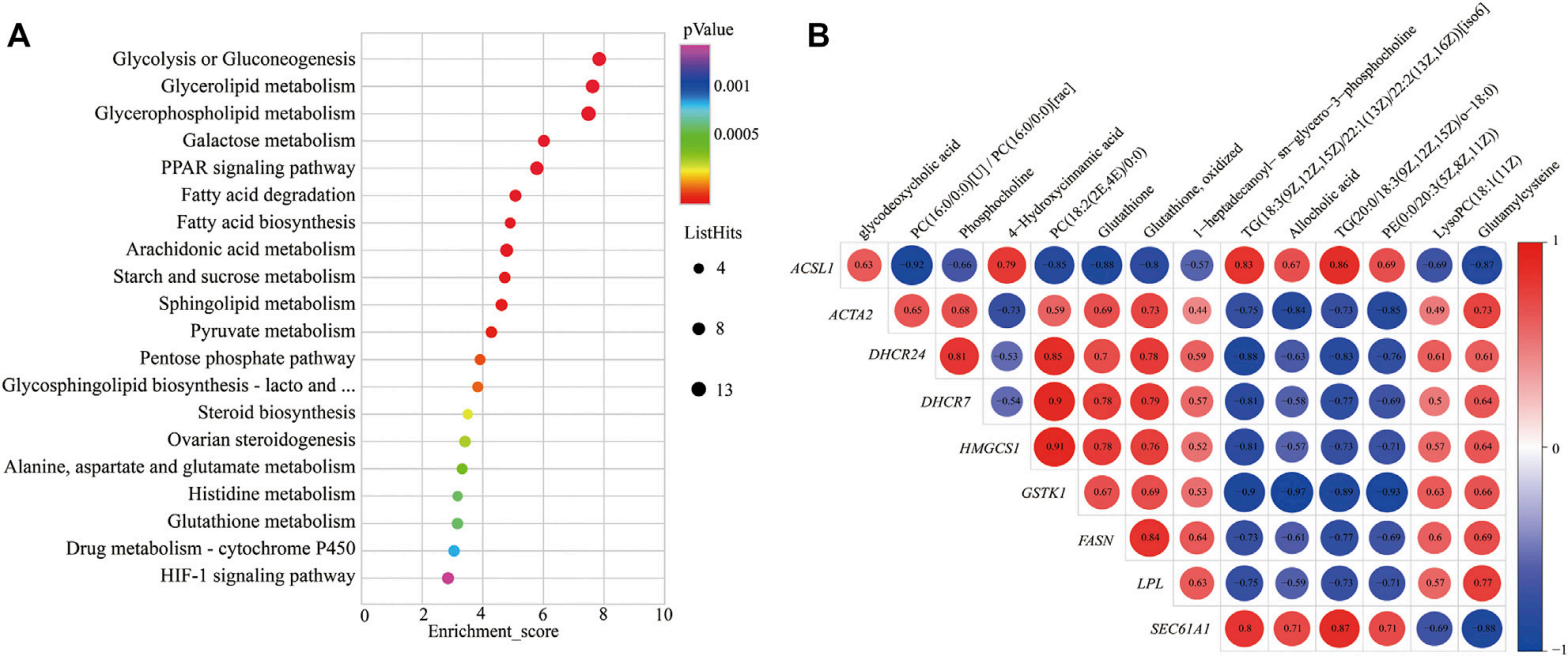
KEGG pathways enrichment and correlation analysis for DEGs and differential metabolite–related energy metabolism. **(A)** KEGG pathways enrichment for DEGs and differential metabolite–related energy metabolism. YC group versus YW group. **(B)** Correlation analysis of the differential metabolites and the DEG-related energy metabolism. *Y* axis represents different genes. *X* axis represents the different metabolites. Color intensity indicates the following: red: positively correlated; blue: negatively correlated.

To further study the potential relationship between DEGs and metabolites. The metabolomic and transcriptomic data were combined and analyzed by MetaboAnalyst 5.0. As shown in Figure 4A, the results demonstrated that the pathways, such as glycolysis or gluconeogenesis, PPAR signaling pathway, fatty acid degradation, fatty acid biosynthesis, arachidonic acid metabolism, pyruvate metabolism, pentose phosphate pathway, steroid biosynthesis, alanine, aspartate, and glutamate metabolism, glutathione metabolism, drug metabolism—cytochrome P450, and HIF-1 signaling pathway, were significantly enriched. Indicating energy metabolism plays an important role in yaks to adapt to nutritional stress due to a shortage of forage. Meanwhile, correlation analysis was performed to examine the association of differential metabolites with DEGs. The correlation analysis between DEGs and differential metabolites related to energy metabolism also showed that PC (18:2 (2E, 4E)/0:0), phosphocholine, glutathione, and glutamylcysteine were positively correlated with DHCR24, DHCR7, HMGCS1, LPL, FASN, ACAT2, and GSTK1, whereas glycodeoxycholic acid, TG (20:0/18:3 (9Z, 12Z, 15Z)/o-18:0), and 4-hydroxycinnamic acid displayed negative correlation (Figure 4B).

## Discussion

The present study revealed a potential mechanism of yak adaption to the long-term withered grass period by regulating hepatic energy metabolism to alter related metabolite and gene pathways to reduce fat catabolism as possible, to maintain the energy homeostasis of whole-body in the yak, further coping with a shortage of forages and adapting to the extreme environment of the QTP.

With the extension of the cold season, the content of CP and EE in herbage decreased, and that of PDF and ADF increased in a previous study (Saul et al., 2009; Zhou et al., 2020), which is consistent with our research results. In this present, compared within a warm season, the content of DM and ADF increased in the cold season, whereas the content of CP and EE in herbage decreased. Therefore, it could be inferred that the BW loss during the cold season resulted from low nutrient contents in forage, which were insufficient to meet the maintenance requirement of growing yaks during the cold season. Meanwhile, the levels of GLU, NEFAs, INS, and IGF-1 in the blood can reflect the energy metabolism of the animal body. Studies have shown that the concentrations of GLU and NEFAs in serum are regulated by the hormones GH, INS, and IGF-1, which play an important role in the synthesis, decomposition, and utilization of sugars and lipids (McGillicuddy et al., 2009). Results of the present study suggest that the levels of glucose (GLU) were remarkably decreased in the cold season; this may be due to when yaks are in a state of starvation or hypoxia, the energy produced by glucose from grass is unable to maintain the normal physiological function of yak, and the demand for glucose exceeds the gluconeogenesis in the liver, and the concentration of GLU decreases accordingly (Giulia Esposito et al., 2014). Previous studies have found that in dairy cows, serum IGF-1 concentration increased with an increase in nutrient intake, whereas the concentration of GH decreased with an increase in nutrient intake (Weller et al., 2016). This experimental result also found that the serum IGF-1 concentration was observably lower in the cold season than in the warm season. Meanwhile, the concentration of GH was significantly higher in the cold season. The possible reasons may be the abundant grass in the warm season, the average daily gain of the yak has been improved, and the growth and development of the animal body tend to mature with the increase in ADG, which gradually reduces the amount of protein synthesis in the body and increases fat deposition. Therefore, the role of GH is relatively weakened, and the serum GH concentration also decreases (Firmenich et al., 2020). Serum triglyceride, total cholesterols, LDL, and HDL reflect the status or rate of lipid metabolism (Huang et al., 2013). Compared with the warm season in this study, the concentration of CH, HDL-C, and LDL-C was significantly decreased in the cold season, implying that the synthesis of lipid metabolism was inhibited during the cold season. Also, INS and LPL were remarkably lower in the cold season than in the warm season; a report from the literature indicated that plasma INS levels were low during energy restriction in most species (Radcliff et al., 2004), consistent with our results, indicating that gluconeogenesis was promoted by the INS signaling pathway to maintain glucose homeostasis during the shortage of forage (Feng et al., 2020). Moreover, our previous study also showed that in the yak, the levels of INS and LPL were significantly higher in the warm season those that in the cold season, which may be used for compensatory growth involved in the supplement of extrahepatic lipid depots and maintenance of body energy of yak in the cold season (Ding et al., 2012).

In the present study, we evaluated hepatic metabolomic alterations through different seasons using LC-MS and found that 306 metabolites were significantly altered between the YC group and the YW group (VIP > 1, *p*<0.05, and FC ≤ 0.67 or ≥1.5), of which 213 metabolites were up-regulated, and 93 metabolites were down-regulated in the cold season. A most important finding was that glutathione and PC (18:2 (2E, 4E)/0:0) were remarkably decreased in the YC group, whereas TG increased. Glutathione is a tripeptide that plays a pivotal role in reducing oxidative stress, maintaining redox balance, enhancing metabolic detoxification, and regulating immune system function (Minich and Brown, 2019). PC, a glycerophospholipid and principal component of the VLDL monolayer, is the major phospholipid component of all plasma lipoprotein classes (Cole et al., 2012), which may help in lipid transport and metabolism and boost VLDL synthesis in the liver of yak (Zhou et al., 2020), indicating that a decrease in the levels of circulating VLDLs and HDLs (Cole et al., 2012; Mcfadden, 2020) was in agreement with the low levels of LDL and HDL in the serum; these results implied free fatty acids as triglyceride for tissues as energy source storage for utilization (Hocquette et al., 1998). Of note, we also observed a significant decrease in metabolites involved in glutathione metabolism, implying that the shortage of forage may increase yak exercise and physical activity, thereby inducing oxidative stress. This result agrees with Godin and Wohieb’s findings, which suggested that a strong ultraviolet and low oxygen environment on the Qinghai Tibet Plateau would increase the formation of free radicals in animals. In contrast, oxidative stress was increased under nutritional deficiency (Godin and Wohieb, 1988).

The adaptive response to food deprivation is associated with major transcriptional and metabolic alterations; one of the most major metabolic changes observed during starvation is increased lipid catabolism in the liver (Settembre et al., 2013). In this study, the gene expression profiles showed that DEGs were significantly enriched in the bile secretion, steroid biosynthesis, PPAR signaling pathway, and fatty acid biosynthesis. In these pathways, *ACSL6* and *ACSL1* were up-regulated, whereas LDLR, HMGCR, IGF1R, DHCR7, FABP4, SCD, HMGCS1, SLC27A2, HKDC1, LPL, OLR1, ACACA, ACACB, and FASN were down-regulated in the YC group. The ACC-α (ACC1/ACACA), which catalyzes the carboxylation of acetyl-CoA to malonyl-CoA, is a key rate-limiting enzyme of DNL synthesis in the mammalian cytosol (Ma et al., 2011). The ACC-β (ACC2/ACACB) is a regulator of mitochondrial fat oxidation, and ACACB knockout in mice has reportedly increased fat oxidation and total energy expenditure and reduced fat mass (Ma et al., 2011). FASN and SCD are important lipogenic enzymes, and changes in their activities can change the biosynthesis rate of fatty acids (Cogburn et al., 2020). The LPL is the major enzyme responsible for hydrolyzing triglycerides present in the triglyceride-rich lipoproteins VLDL and CMs to provide free fatty acids for tissue utilization or storage (Kersten, 2014; Nyrén et al., 2019). Oxidized low-density lipoprotein receptor 1 (OLR1) is one of the most vital lipoprotein receptors regulating fat deposition, which overexpression could augment free fatty acid uptake and cholesterol content (Chui et al., 2005). Solute carrier family 27 member 2 (SLC27A2) is a transmembrane protein, which plays a crucial in fatty acid degradation and lipid biosynthesis (Caimari et al., 2010). Moreover, our previous study in yak showed the expression of genes FASN, LPL, ACACA, PPARγ, and SREBP-1c with increasing energy levels during the cold season, whereas there was low expression of HSL, CPT-1, and ATGL (Yang et al., 2020). Similar to our results, in comparison with the warm season, the expressions of genes responsible for *de novo* fatty acids synthesis (ACACA, ACACB, and FASN), fatty acid uptake (LPL, OLR1), the rate-limiting steps of fatty acid uptake (SLC27A2), FA desaturation (SCD), and FA transportation (LDLR) were significantly down-regulated in the liver of cold season grazing yak, indicating that malnutrition reduced the capacity of liver for synthesis, and degradation of *de novo* fatty acid and FA uptake as well as the transportation of fatty acids from the liver to other parts of the body in the form of VLDL. It may be used for compensatory growth and to maintain the body’s energy homeostasis, thereby coping with the harsh conditions of the QTP (Ding et al., 2012). This speculation was further supported by the serum levels of LDL-C, LPL, and total cholesterol, which were remarkably lower for the YC group than the YW group. And also consistent with the study by Vahmani et al. (2014), who reported that the genes involved in lipid synthesis, including ACACA, FASN, LPL, SCD1, FADS1, and FADS2, were down-regulated under nutritional deficiency.

Long-chain acyl-CoA synthetases (ACSL 1–6) are key enzymes regulating the partitioning of acyl-CoA species in lipid metabolism and take part in lipid synthesis or β-oxidation (Ellis et al., 2010). ACSL1 plays a key role in the synthesis of triglycerides, phospholipids, and cholesterol esters. It was reported that high expression of ACSL1 reduced fatty acid β-oxidation through the PPARγ pathway, further increasing triglyceride levels (Li Y. et al., 2020). Fasting and exercise decreased the expression levels of ACSL6 and other lipid synthesis genes (Teodoro et al., 2017). We observed in the liver the ACSL1, and the ACSL6 gene was up-regulated in the cold season, which was not in agreement with previous results. Previous research indicated in rat fasting up-regulated ACSL1 and ACSL4 mRNA expression levels and down-regulated expression of ACSL6 (Mashek et al., 2006). The possible reasons may be that ACSL6 was regulated by INS and speculated that biological circumstances that promote increased INS sensitivity would be corrected with higher levels of ACSL6 in the liver (Teodoro et al., 2017).

Cholesterol homeostasis in the liver of mammals is maintained through exogenous absorption, endogenous synthesis, and excretion or conversion of cholesterol into bile acids. A reciprocal relationship between these processes regulates circulating cholesterol levels in response to dietary interventions (Liu et al., 2017; Oczkowicz et al., 2020). Bile acid, a steroid acid synthesized in the liver, is responsible for fat metabolisms, such as digestion and absorption (Li R. et al., 2020). A previous study has shown that lipid metabolism (particularly cholesterol and steroid metabolism) was significantly increased with increasing dietary forage levels and the genes (HMGCS1, HMGCR, MSMO1, and DHCR7) enriched in the related pathways (Shi et al., 2018). Similar to our results, in the current study, the GO and KEGG enrichment analysis found that DEGs (HMGCR, HMGCS1, and DHCR7) involved in the cholesterol biosynthetic process, the bile secretion, and sterol metabolism were significantly down-regulated in the absence of forage. Decreased cholesterol synthesis, steroid biosynthesis, and bile secretion in the liver might be responsible for the increased energy utilization and adaption to a harsh QTP environment in the cold season (Miron and Tirosh, 2019).

Meanwhile, we also observed that glutathione metabolism was significantly involved in liver transcriptome and metabolome of grazing yaks in the cold season. The glutathione S-transferases (GSTs) are a superfamily of isoenzymes that play important roles in the diminution of antioxidant injury, immune system function regulation, signaling pathways, and enhancement of metabolic detoxification. It has been demonstrated that GSTM3 and GSTK1 contributed to oxidative stress–mediated liver damage (Uno et al., 2020). We observed that down-regulation of GSTM3 and GSTK1 expression in the liver of cold season grazing yaks, which may be due to shortage of forage and low temperature in the cold season, induced excessive physical activity and negative nutrient balance, thereby inducing oxidative stress and immune response (Ren et al., 2019).

**FIGURE 5:**
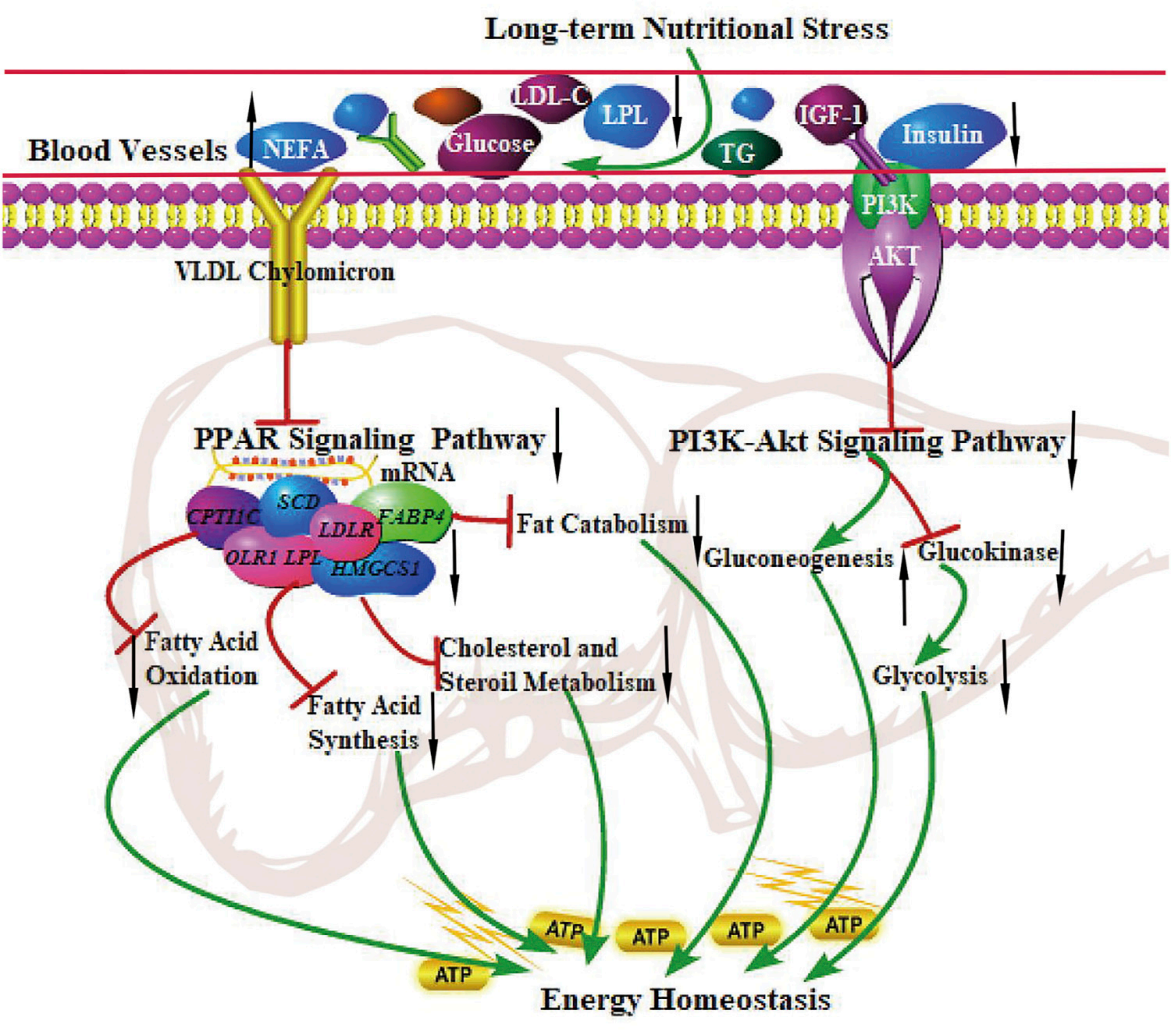
The regulation of the PPAR signaling pathway and PI3K-Akt signaling pathway in the liver of the yak under long-term nutritional stress. ↑: up-regulation of genes expression or enhanced the pathways; ↓: down-regulation of genes expression or diminished the pathways. →: promote or result in. ⊥: the genes expression or metabolic pathways were inhibited.

The PPAR signaling pathway is vital in metabolism, lipolysis, adipogenesis, angiogenesis, INS sensitivity, inflammatory response, and cell growth. A recent study showed that the PPAR signaling pathway (related to lipolysis) was up-regulated in grazing conditions (Laporta et al., 2014; Vahmani et al., 2014; Ren et al., 2019). Strangely, The finding from the present study was the observation that DEGs were significantly enriched in the PPAR signaling pathway, the genes related to fatty acid uptake (LPL, OLR1), adipogenesis (SCD, FASN, and FADS2), lipolysis (FABP4), the rate-limiting steps of fatty acid uptake (SLC27A2), and fatty acid oxidation (CPT1C) were all down-regulated, indicating the inhibition of fatty acid synthesis, adipogenesis, fat catabolism, and fatty acid oxidation in grazing of the cold season. It is not consistent with the results of hepatic metabolism in other ruminants. In addition, the PI3K-Akt signaling pathway plays an essential role in regulating hepatic glucose homeostasis and INS sensitivity. PI3K activates Akt and accelerates the phosphorylation of PDK1, thereby suppressing hepatic gluconeogenesis and accelerating glycogen synthesis (Pitaloka et al., 2019). Our results showed that the PI3K-Akt signaling pathway was significantly inhibited in the cold season, implying that the decreased gene expression levels involved in the PI3K-Akt pathway may enhance hepatic glycogenolysis and gluconeogenesis to maintain energy homeostasis of the body (Rui, 2014). The study reported that compared with small-tailed Han sheep, Tibetan sheep had higher gluconeogenesis and ketogenesis in the liver in negative energy balance to cope with low energy intake and regulate whole-body energy homeostasis under the harsh environment of the QTP (Jing et al., 2021). These physiological characteristics in yak are similar to those in Tibetan sheep, presenting low fatty acid oxidation and fat catabolism and high gluconeogenesis to maintain whole-body energy homeostasis under grazing conditions in the cold season. The regulatory mechanism of the PPAR signaling pathway and PI3K-Akt signaling pathway in the liver of yak under long-term nutritional stress is shown in Figure 5.

In addition, the correlation analysis between DEGs and differential metabolites related to energy metabolism showed that PC, phosphocholine, glutathione, and glutamylcysteine were positively correlated with DHCR24, DHCR7, HMGCS1, LPL, FASN, ACAT2, and GSTK1. At the same time, TG displayed a negative correlation; this may be due to yak to cope with the shortage of forages and maintain the energy homeostasis of whole-body during the cold season; the LPL catalyzes the hydrolysis of VLDL and CMs to provide free fatty acids for tissue utilization. This process increased the metabolite TG. It decreased the expression of genes of LPL and VLDL. PC is a principal component of the VLDL monolayer and helps lipid transport metabolism and boost VLDL synthesis in the liver of yak (Zhou et al., 2020). Therefore, the low expression of the VLDL causes to decrease in the metabolite PC and thereby inhibits lipid metabolism. So, it was speculated that the decrease in LPL may affect the production of metabolite PC and expression of genes LPL and VLDL, which further affects the expression of genes FASN, DHCR24, DHCR7, HMGCS1, and ACAT2.

## Conclusion

The study investigated the potential mechanism of yak adaption to the long-term withered grass period by transcriptomics and metabolomics under natural grazing. The metabolome and transcriptome analysis showed that nutritional stress caused differential alterations of various hepatic metabolites, genes and related pathways, such as glycolysis or gluconeogenesis, lipid metabolism (particularly fatty acid, cholesterol, and steroid metabolism), and glutathione metabolism. But most importantly, the reduced fatty acid synthesis, fatty acid oxidation, adipogenesis, and fat catabolism facilitated gluconeogenesis by regulating the PPAR signaling pathway and PI3K-Akt signaling pathway to maintain the energy homeostasis of whole body in the yak, thereby coping with the shortage of forages and adapting to the extreme environment of the QTP.

## Data Availability

The datasets presented in this study can be found in online repositories. The names of the repository/repositories and accession number(s) can be found in the article/Supplementary Material.

## Glossary

ACACB: Acetyl-CoA carboxylase β
ACACA: Acetyl-CoA carboxylase α
ADF: acid detergent fiber
ADFD: acid detergent fiber digestibility
AMPK: adenosine 5′-monophosphate-activated protein kinase
ACSL1: Long-chain Acyl-CoA Synthetases 1
ACSL6: Long-chain Acyl-CoA Synthetases 6
BW: body weight
CH: cholesterol
CK: creatine kinase
CPD: crude protein digestibility
CPT1C: Carnitine palmitoyltransferase 1C
CP: crude protein
CM: chylomicrons
DNL: *de novo* lipogenesis
DM: the dry matter
DMI: dry matter intake
DMD: dry matter digestibility
DEGs: differential expression genes
EE: ether extract
FASN: fatty acid synthase
FAS: Fatty acid synthetase
FDR: false discovery rate
FABP4: fatty acid binding protein 4
GH: growth hormone
GSH-PX: Glutathione peroxidase
GLU: glucose
GO: Gene Ontology
GSTM3: Glutathione S-transferases Mu 3
GSTK1: Glutathione S-transferase kappa 1
HDL-C: high-density lipoprotein cholesterol
HSL: Hormone-sensitive fatty lipase
IGF-1: insulin-like growth factor-1
IGF-2: insulin-like growth factor-2
INS: insulin
KEGG: Kyoto Encyclopedia of Genes and Genomes
LPL: lipoprotein lipase: lipoprotein lipase
LPL: lipoprotein lipase: lipoprotein lipase
LDH: Lactate dehydrogenase
LDL-C: low-density lipoprotein cholesterol
MEI: metabolic energy intake
NDFD: neutral detergent fiber digestibility
NEFAs: nonesterified fatty acids
NDF: neutral detergent fiber
MDA: malondialdehyde
OLR1: Oxidized low-density lipoprotein receptor 1
OPLS-DA: Orthogonal partial least-squares discriminant analysis
PCA: principal component analysis
PC: Phosphatidylcholine
QTP: Qinghai-Tibetan Platea
RIN: RNA Integrity Number
SOD: Superoxide dismutase
SCD: stearoyl-CoA desaturase
SLC27A2: solute carrier family 27 member 2
TG: triglycerides
T-AOC: Bovine total antioxidant capability
VIP: variable importance in the projection
YC: cold season group
YW: warm season group
