# Supplementary material for: Transcriptomic and Metabolomic Analyses Reveal Inhibition of Hepatic Adipogenesis and Fat Catabolism in Yak for Adaptation to Forage Shortage During Cold Season
Source: Front Cell Dev Biol. 2022 Jan 17;9:759521. doi: 10.3389/fcell.2021.759521 (PMC8802892; doi:10.3389/fcell.2021.759521)

**Supplementary Table 1:** The nutritional composition of pasture herbage

**Supplementary Table 2:** Intake and nutrient digestibility of yak in different seasons

**Supplementary Table 3:** Primers of genes selected for qRT-PCR

**Supplementary Figure1:** The volcanic map of different metabolites in liver of YW and YC groups. Grey was the non-significant difference gene, red and blue was the significant difference metabolites. The X-axis represented log2 Fold Change, and the Y-axis represented -log10 P-value.

**Supplementary Figure 2:** Validation of sequencing data by qPCR. X-axis represents 10 DEGs were randomly selected. Y-axis represents the expression level of DEGs, which is showed by log2FoldChange.

**Supplementary Table 1**

| Item | Treatment | | SEM | *P*-value |
| --- | --- | --- | --- | --- |
|  | YC | YW |  |  |
| Dry matter (DM) | 94.34 | 92.74 | 0.57 | 0.033 |
| Crude protein (CP) | 4.17 | 8.25 | 1.44 | ＜0.001 |
| ether extract (EE) | 5.10 | 7.46 | 0.84 | 0.014 |
| neutral detergent fiber (NDF) | 63.50 | 58.03 | 1.93 | ＜0.001 |
| acid detergent fiber (ADF) | 31.91 | 29.61 | 0.82 | 0.012 |

**Supplementary Table 2**

| Item | Treatment | | SEM | *P*-value |
| --- | --- | --- | --- | --- |
|  | YC | YW |  |  |
| dry matter intake (DMI) | 7.44 | 10.34 | 1.03 | ＜0.001 |
| metabolic energy intake (MEI) | 6.76 | 8.94 | 0.77 | 0.013 |
| dry matter digestibility (DMD) | 66.33 | 71.00 | 1.64 | 0.001 |
| crude protein digestibility (CPD) | 70.21 | 80.08 | 3.49 | ＜0.001 |
| neutral detergent fiber digestibility (NDFD) | 60.18 | 71.79 | 4.10 | 0.001 |
| acid detergent fiber digestibility (ADFD) | 46.22 | 63.21 | 6.15 | ＜0.001 |

**Supplementary Table 3**

| Genes | Accession |  | Primer sequence (5’-3’) | Product size |
| --- | --- | --- | --- | --- |
| β-actin | XM_005887322.2 | F | ACCATCGGCAATGAGCG | 150bp |
|  |  | R | CACCGTGTTGGCGTAGAG |  |
| HKDC1 | [XM_005906353.1](https://www.ncbi.nlm.nih.gov/nuccore/XM_005906353.1) | F | CGGAAACCTCTGCTCACCTC | 119bp |
|  |  | R | GGAACCTGTCCACCTTCTTGA |  |
| HK2 | [XM_005906747.2](https://www.ncbi.nlm.nih.gov/nuccore/XM_005906747.2) | F | CCTGGCAGACAGAGGTTTGA | 137bp |
|  |  | R | ATTCCCCTTGTCTTGAGCCG |  |
| ACAT2 | [XM_005900391.2](https://www.ncbi.nlm.nih.gov/nuccore/XM_005900391.2) | F | CCCAGCTAATGCTTCAGGAAT | 128bp |
|  |  | R | GTTCCACATCTGCTTGTGCC |  |
| ACSL1 | [XM_005906554.2](https://www.ncbi.nlm.nih.gov/nuccore/XM_005906554.2) | F | TGCTGCCTGACTATTGCTGG | 136bp |
|  |  | R | CTTTCACACATACCTCGCCCT |  |
| ACACB | [XM_005891509.1](https://www.ncbi.nlm.nih.gov/nuccore/XM_005891509.1) | F | CATGTACGGCCACCAGTTCT | 139bp |
|  |  | R | CCGCACATAGACCTCCAAGG |  |
| ALDOC | [XM_005888186.2](https://www.ncbi.nlm.nih.gov/nuccore/XM_005888186.2) | F | AGATGAGTCTGTAGGCAGCA | 112bp |
|  |  | R | CACACGGTCATCGGCACTAA |  |
| ACSL6 | [XM_014480857.1](https://www.ncbi.nlm.nih.gov/nuccore/XM_014480857.1) | F | GACAGAGGGCAAGAGTGTGG | 148bp |
|  |  | R | CTTTTGGGTTCCCTGTCGTG |  |
| SEC61A1 | [XM_014476766.1](https://www.ncbi.nlm.nih.gov/nuccore/XM_014476766.1) | F | TCATCTATTTCCAGGGCTTCCG | 143bp |
|  |  | R | GTACAGGTTGGACACGAGGG |  |
| SDS | [XM_005904739.2](https://www.ncbi.nlm.nih.gov/nuccore/XM_005904739.2) | F | AGCGCAGGCTATGAAGGTTT | 110bp |
|  |  | R | CCAGGATCTTCTCATCATCCACA |  |
| GMPPB | [XM_005892095.2](https://www.ncbi.nlm.nih.gov/nuccore/XM_005892095.2) | F | CGCAGGAGCAAAAGCTAGGAAT | 107bp |
|  |  | R | TGCAGTCTCACAGAGCAAGT |  |

**Supplementary Figure 1**

**
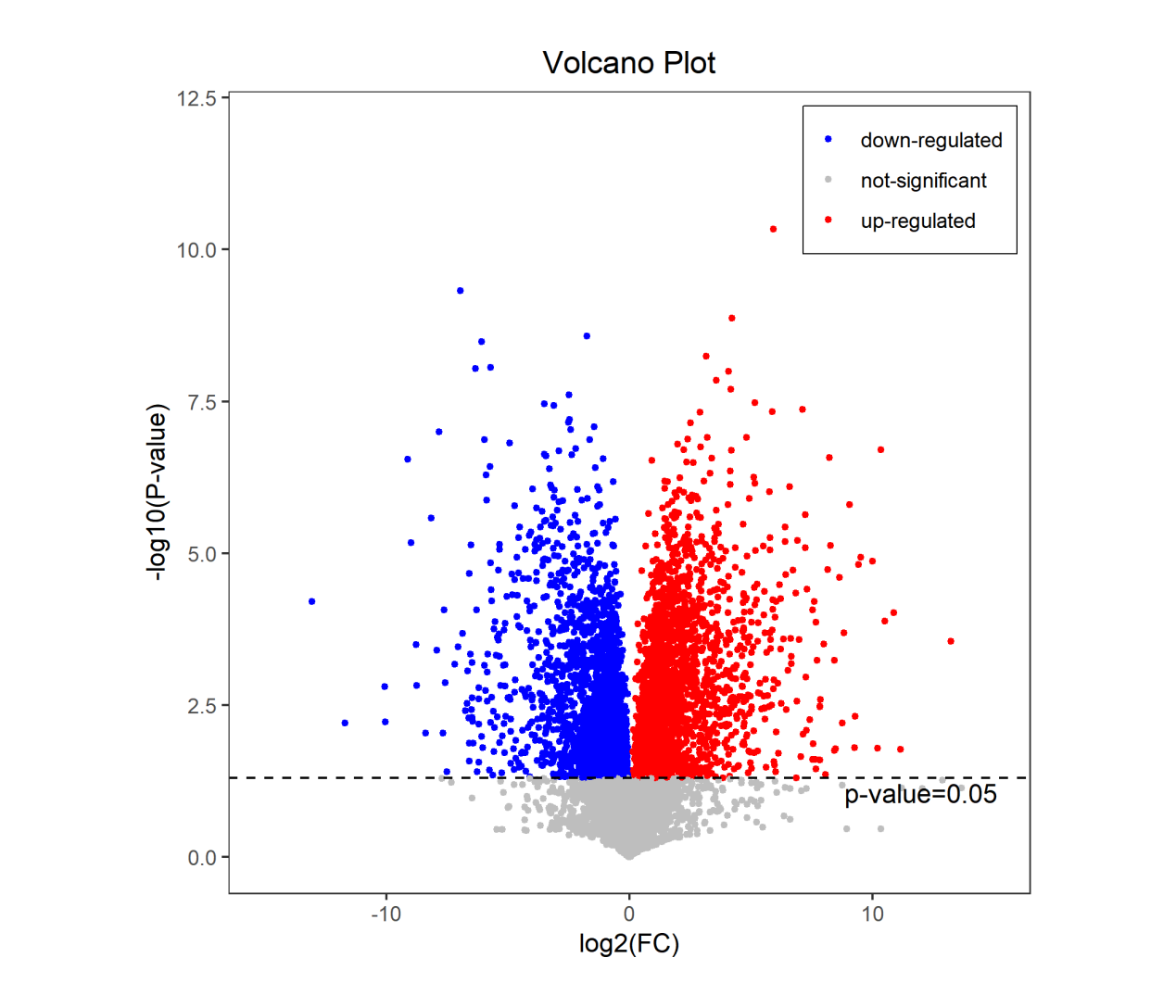
**

**Supplementary Figure 2**


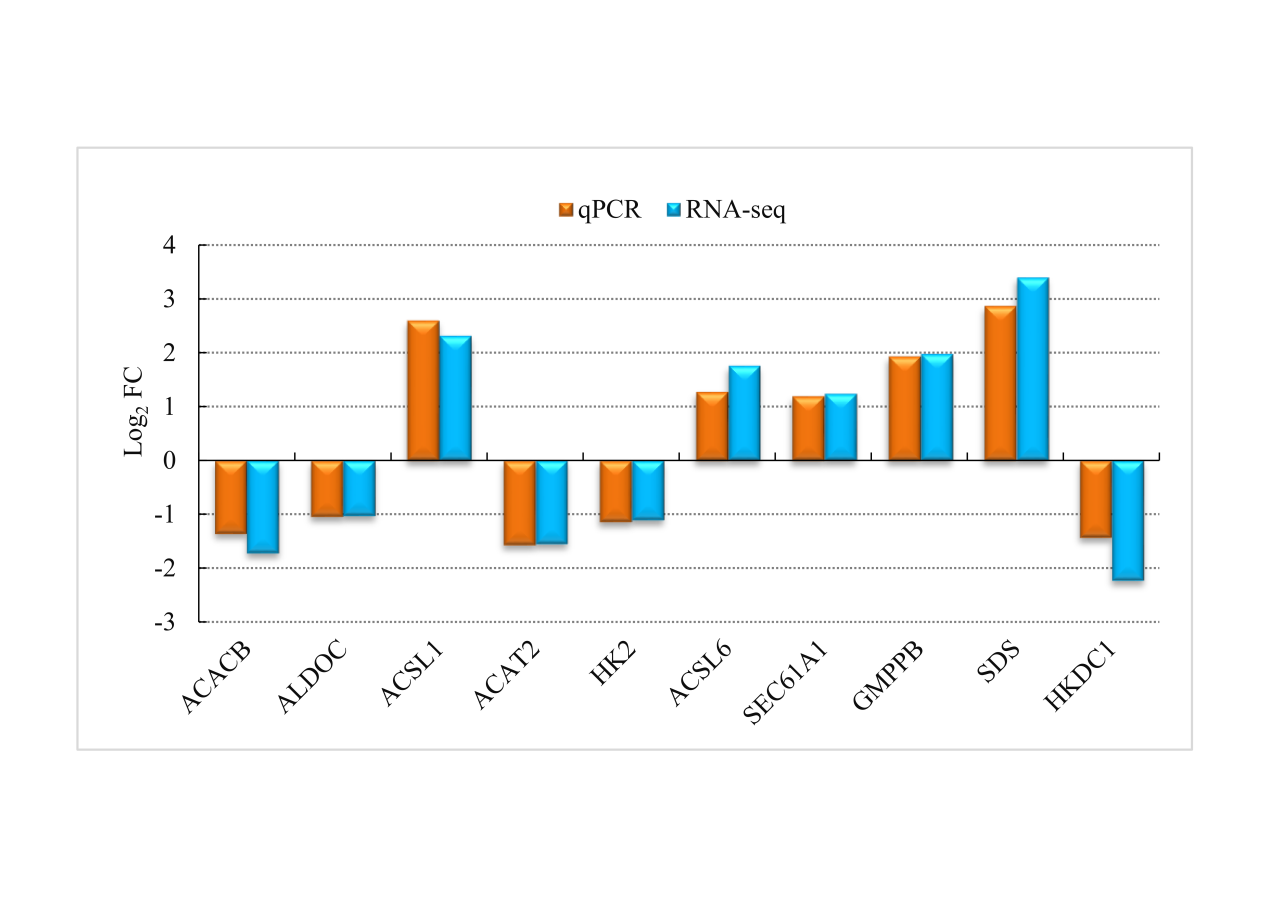

Supplement: Supplementary file 1 [file DataSheet1.zip › Supporting Information/SupplementaryTables and Figures.docx]
